# Fracture toughness and structural evolution in the TiAlN system upon annealing

**DOI:** 10.1038/s41598-017-16751-1

**Published:** 2017-11-28

**Authors:** M. Bartosik, C. Rumeau, R. Hahn, Z. L. Zhang, P. H. Mayrhofer

**Affiliations:** 1 Institute of Materials Science and Technology, TU Wien, A-1060 Vienna, Austria; 2 Erich Schmid Institute of Materials Science, Austrian Academy of Sciences, A-8700 Leoben, Austria

## Abstract

Hard coatings used to protect engineering components from external loads and harsh environments should ideally be strong and tough. Here we study the fracture toughness, *K*_IC_, of Ti_1−x_Al_x_N upon annealing by employing micro-fracture experiments on freestanding films. We found that *K*_IC_ increases by about 11% when annealing the samples at 900 °C, because the decomposition of the supersaturated matrix leads to the formation of nanometer-sized domains, precipitation of hexagonal-structured B4 AlN (with their significantly larger specific volume), formation of stacking faults, and nano-twins. In contrast, for TiN, where no decomposition processes and formation of nanometer-sized domains can be initiated by an annealing treatment, the fracture toughness *K*_IC_ remains roughly constant when annealed above the film deposition temperature. As the increase in *K*_IC_ found for Ti_1−x_Al_x_N upon annealing is within statistical errors, we carried out complementary cube corner nanoindentation experiments, which clearly show reduced (or even impeded) crack formation for annealed Ti_1−x_Al_x_N as compared with their as-deposited counterpart. The ability of Ti_1−x_Al_x_N to maintain and even increase the fracture toughness up to high temperatures in combination with the concomitant age hardening effects and excellent oxidation resistance contributes to the success of this type of coatings.

## Introduction

Hard coatings are applied to protect tool and component surfaces as well as entire devices in harsh environments and/or demanding application conditions. The coatings are usually ceramic materials, which are known for their beneficial properties such as high hardness and wear resistance, high melting temperatures, high-temperature strength, chemical inertness and oxidation resistance. However, these materials often possess a relatively low (fracture) toughness. A certain degree of toughness, however, is crucial for the reliability and safe operation of critical components. Various strategies have been applied to enhance the fracture toughness of bulk materials^1^ and hard coatings^2,3^.

Since the pioneer works in the nineteen eighties^4,5^, Ti_1−x_Al_x_N has evolved to one of the most widely used and industrial relevant hard coating systems^6^. Age hardening effects are (besides enhanced oxidation resistance^7^ and resistance against wear^4,5^ compared to TiN) considered to be the major basis for its industrial success. At temperatures typical for cutting tools operation, supersaturated face-centered cubic Ti_1−x_Al_x_N isostructurally decomposes into nanometer-sized AlN-rich and TiN-rich domains. This is due to spinodal decomposition causing self-hardening effects^8–11^. Nonetheless, the influence of its characteristic thermally activated decomposition and the resulting self-organized nanostructure on the fracture toughness is yet to be studied.

The present work revolves around the hypothesis that (besides the well-known self-hardening effects^9^) also the fracture toughness of Ti_1−x_Al_x_N coatings increases at elevated temperatures. Potential fracture toughness enhancing mechanisms in the self-organized nanostructure of B1 AlN-rich and TiN-rich domains^8–11^ are based on: coherency strains, spatially fluctuating elastic properties, and stress-induced phase transformation toughening from cubic to hexagonal AlN phases under volume expansion at the tip of a propagating crack similar to Yttrium-stabilized zirconia bulk ceramics^12^ or Zr-Al-N based nanoscale multilayers^13^. We will also see that the B4 AlN phase formation can play a key role for the fracture toughness evolution of Ti_1−x_Al_x_N. By using high-resolution transmission electron microscopy (HRTEM), we observed severely distorted B4 AlN with multiple stacking faults and indications of nano-twins. Twinning represents a mechanism capable of simultaneously enhancing strength and ductility in materials^14^.

We carried out cantilever deflection (and cube corner nanoindentation experiments) to study the evolution of the fracture toughness of up to 1000 °C *ex-situ* vacuum annealed Ti_1−x_Al_x_N free-standing films and correlated them with the film structural evolution and the mechanical properties, hardness (*H*) and Young’s modulus (*E*), obtained from independent experiments. The mechanical properties were corroborated with HRTEM investigations to give atomic scale insights into the thermally decomposed Ti_1−x_Al_x_N structure. TiN coatings are used as a benchmark, as no decomposition processes are active that would lead to the formation of new nm-sized domains.

## Results

### Structural evolution

**Figure 1:**
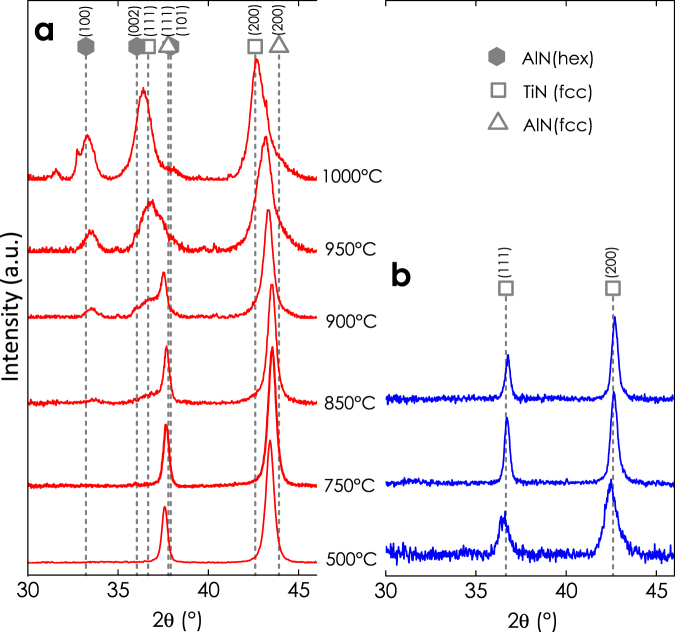
XRD patterns of as-deposited and vacuum annealed (**a**) Ti_0.40_Al_0.60_N and (**b**) TiN films on Al_2_O_3_ $(1\bar{1}02)$ substrates. (JCPDF files: 38–1420 TiN, 25–1495 fcc AlN, 25–1133 hex. AlN). The graphs show the thermally activated decomposition sequence of cubic TiAlN into iso-structural TiN-rich and cubic AlN-rich domains followed by the formation of wurtzite AlN. Annealing of TiN is characterized by recovery of built-in growth defects.

Energy dispersive X-ray spectroscopy (EDXS) analysis rendered a chemical composition of Ti_0.40_Al_0.60_N. Due to the specific sputter condition of the Ti_0.5_Al_0.5_ compound target, the coatings prepared are slightly richer in Al than the target for the deposition parameters used^15^. The oxygen content within the coatings is below 1 at.%, as obtained by elastic recoil detection analysis of coatings prepared under comparable conditions^15^. Figure 1a shows the X-ray diffraction patterns of our Ti_0.40_Al_0.60_N films grown onto Al_2_O_3_ $(1\bar{1}02)$ substrates after vacuum annealing at different annealing temperatures, *T*_a_, for 10 min. Up to 750 °C, Ti_0.40_Al_0.60_N maintains its single phase face-centered cubic (rock-salt-type, B1) structure. The slight peak shift to higher 2θ angles and decrease in peak broadening indicate recovery of built-in structural point and line defects, which results in a lattice parameter decrease in the films. The peak shift to higher 2θ angles also suggests B1 AlN formation (its lattice parameter is smaller as compared to Ti_0.40_Al_0.60_N^16^, hence the diffraction peaks occur at higher 2θ angles). Between 850 and 1000 °C, an asymmetric peak broadening is observed, which indicates isostructural formation of cubic AlN- and TiN-rich domains. Especially, the right shoulder in vicinity of the cubic (200) peak – indicative for cubic AlN formation – is clearly visible and becomes more pronounced with increasing temperature. Hexagonal (wurtzite-type, B4 structured) AlN first emerges at 850 °C and its phase fraction increases with increasing temperature. The shift of the XRD reflections from the major cubic structured Ti_1−x_Al_x_N matrix phase to lower 2θ angles is a result of decreasing Al content (hence, the XRD peaks shift towards the lower 2θ position of TiN). On the other hand, compressive stresses, *e.g*., induced by the B1 to B4 phase transformation of AlN^17^ under volume expansion of ~26%^16^ or by thermal stresses, contribute to the peak shift to lower 2θ angles (for the thermal expansion coefficients, α, holds as α_B1-(Ti,Al)N_ > α_Al2O3_ > α_B4-AlN_, see refs^18–20^).

The structural evolution of single phase cubic structured TiN, Fig. 1b, is dominated by recovery of built-in structural point and line defects and results in smaller lattice parameters. Accordingly, the peaks are shifted to larger 2θ angles and become sharper with increasing temperature. Both, Ti_0.40_Al_0.60_N and TiN crystallized in a polycrystalline structure. (For a thorough analysis of the crystallographic texture further investigations would be necessary, *e.g*., pole figure measurements based on X-ray diffraction).

### TEM/HRTEM study

**Figure 2:**
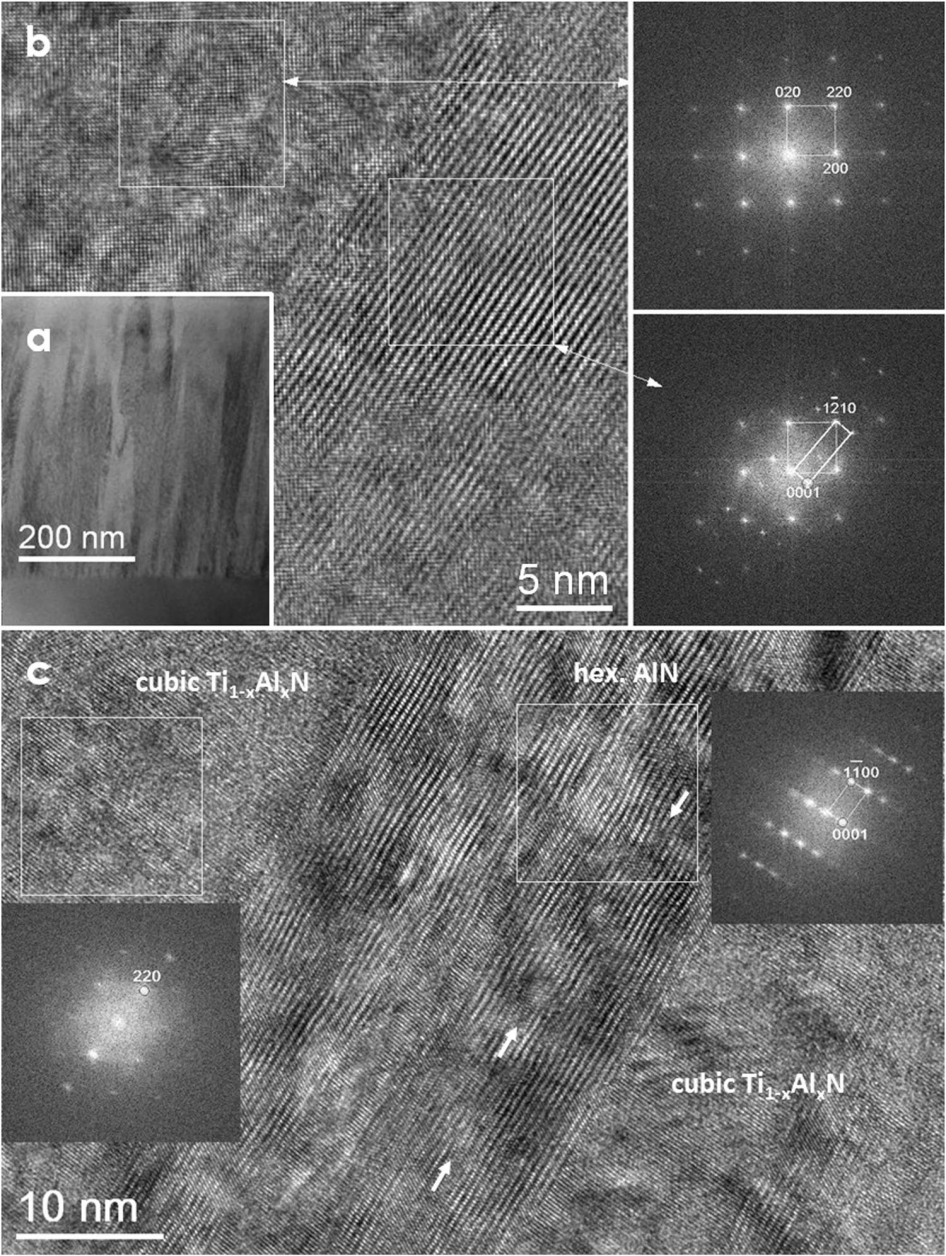
(**a**) Low magnification TEM image showing the columnar morphology of Ti_1−x_Al_x_N; (**b**) HRTEM image covering one cubic Ti_1−x_Al_x_N grain along the [001] zone axis and hexagonal AlN grains along the $[21\bar{1}0]$ direction, the corresponding FFTs are attached; (**c**) HRTEM image showing one hexagonal AlN grain that grows in between two cubic Ti_1−x_Al_x_N grains, viewing direction of AlN is close to hexagonal $[11\bar{2}0]$. Multiple stacking faults and nano-twins in the hexagonal AlN phase are visible. One stacking fault is exemplarily marked with white arrows (pointing to the locations of partial dislocations). Note that all FFTs are obtained from the square regions.

TEM studies were performed on the sample annealed at 900 °C using cross-section samples. A low-magnified image presents an overview of the coating morphology (Fig. 2a), where columnar grains are clearly visible. At this annealing temperature, AlN based hexagonal phases emerge. An atomic resolution TEM image of one portion of grain interfaces are shown in Fig. 2b, the corresponding fast Fourier transforms (FFTs) are seen on the right-hand side. Analysis indicates that a cubic structured Ti_1−x_Al_x_N grain is oriented along the [001] direction while the adjacent hexagonal AlN grain is close to $[21\bar{1}0]$ direction, with an orientation relationship of Ti_1−x_Al_x_N $(2\bar{2}0)$//AlN (0001). This implies that hexagonal AlN (0001) grows on Ti_1−x_Al_x_N $(2\bar{2}0)$ planes with a small misfit of *δ* = $\frac{{d}_{220}^{TiAlN\,}-\,{d}_{1210}^{AlN}\,}{{d}_{220}^{TiAlN}}\,\approx 5.7\, \% \,\,\,$along this direction. The corresponding FFTs clearly signify the plane relationship between these two phases. This has also been proved by tilting the grains to another orientation. Figure 2c shows one hexagonal AlN grain, grown in between two cubic Ti_1−x_Al_x_N grains, viewed along the $[11\bar{2}0]$ direction while Ti_1−x_Al_x_N is off [001] zone axis, as illustrated in the corresponding FFTs (inserted). Here, only a series of planes appear. The orientation relation is Ti_1−x_Al_x_N (220)//AlN $(1\bar{1}00)$ for this case. It is further noted that the planes in hexagonal AlN are severely distorted or inclined which means that internal stress is strongly involved during the phase transformation. There are numerous defects present in the hexagonal AlN regions, for instance stacking faults and nano-twins marked exemplarily with white arrows in Fig. 2c. In some cases, the AlN phase seems to form in the Ti_1−x_Al_x_N matrix, *i.e*. Fig. 2b, since the FFT from AlN contains Ti_1−x_Al_x_N spots. However, hexagonal AlN frequently forms at the grain boundary as demonstrated in Fig. 2c, in which the hexagonal AlN and Ti_1−x_Al_x_N phases are separated and formed in between two Ti_1−x_Al_x_N grains. Consequently, the AlN phase transformation (from cubic to hexagonal) can take place in the matrix and also at the grain boundaries, in agreement with earlier studies^21^.

### Nanoindentation

**Figure 3:**
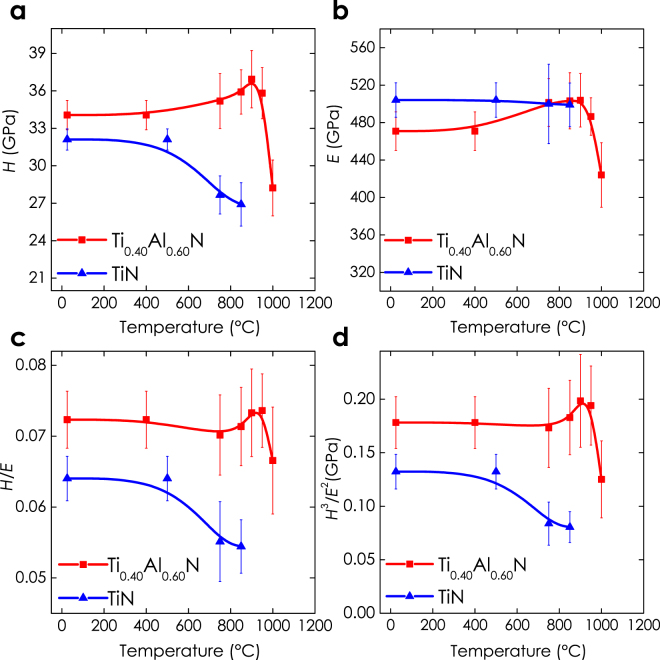
Hardness (*H*), indentation modulus (*E*), (*H*/*E*), and (*H* ^3^/*E* ^2^) ratios of Ti_0.40_Al_0.60_N as a function of annealing temperature (annealing time = 10 min). Age-hardening effects and implications on the toughness criteria are clearly visible.

The mechanical properties as a function of annealing temperature are presented in Fig. 3 and are in line with previous studies reported in literature^9^. The indentation hardness (*H*), Fig. 3a, increases for Ti_0.40_Al_0.60_N (red curves) by ~9% from 34 ± 1 GPa in the as-deposited state to 37 ± 2 GPa at 900 °C, before it decreases again down to 28 ± 2 GPa at 1000 °C. The Young’s modulus (*E*), Fig. 3b, shows a similar trend. Contrarily, the hardness of TiN (blue curves) steadily decreases with increasing *T*_a_, from 32 ± 1 GPa at room temperature to 27 ± 1 GPa at 850 °C, (Fig. 3a), while the Young’s modulus marginally decreases (Fig. 3b). The chosen deposition conditions used in the present study resulted in coatings with excellent mechanical properties in the as-deposited state. In general, age hardening effects are more pronounced for softer coatings, *e.g*., a relative increase of ~25% was observed for Ti_1−x_Al_x_N with an as-deposited hardness of ‘only’ ~26 GPa^21^.

The elastic strain to failure^22–26^, (*H*/*E*), which is often used to qualitatively rate materials for their failure resistance, suggests superior properties of Ti_0.40_Al_0.60_N in comparison with TiN, Fig. 3c. While (*H*/*E*) values for Ti_0.40_Al_0.60_N are maintained up to high temperatures and even increase, the (*H*/*E*) ratio of TiN is below that of Ti_0.40_Al_0.60_N in the as-deposited state and shows a steady decrease upon annealing above the deposition temperature. A similar trend can be observed for the plastic deformation resistance factor^22,25,26^, (*H* ^3^/*E* ^2^), shown in Fig. 3d, indicating superior wear resistance of Ti_0.40_Al_0.60_N in comparison with TiN.

### Micromechanical Testing

**Figure 4:**
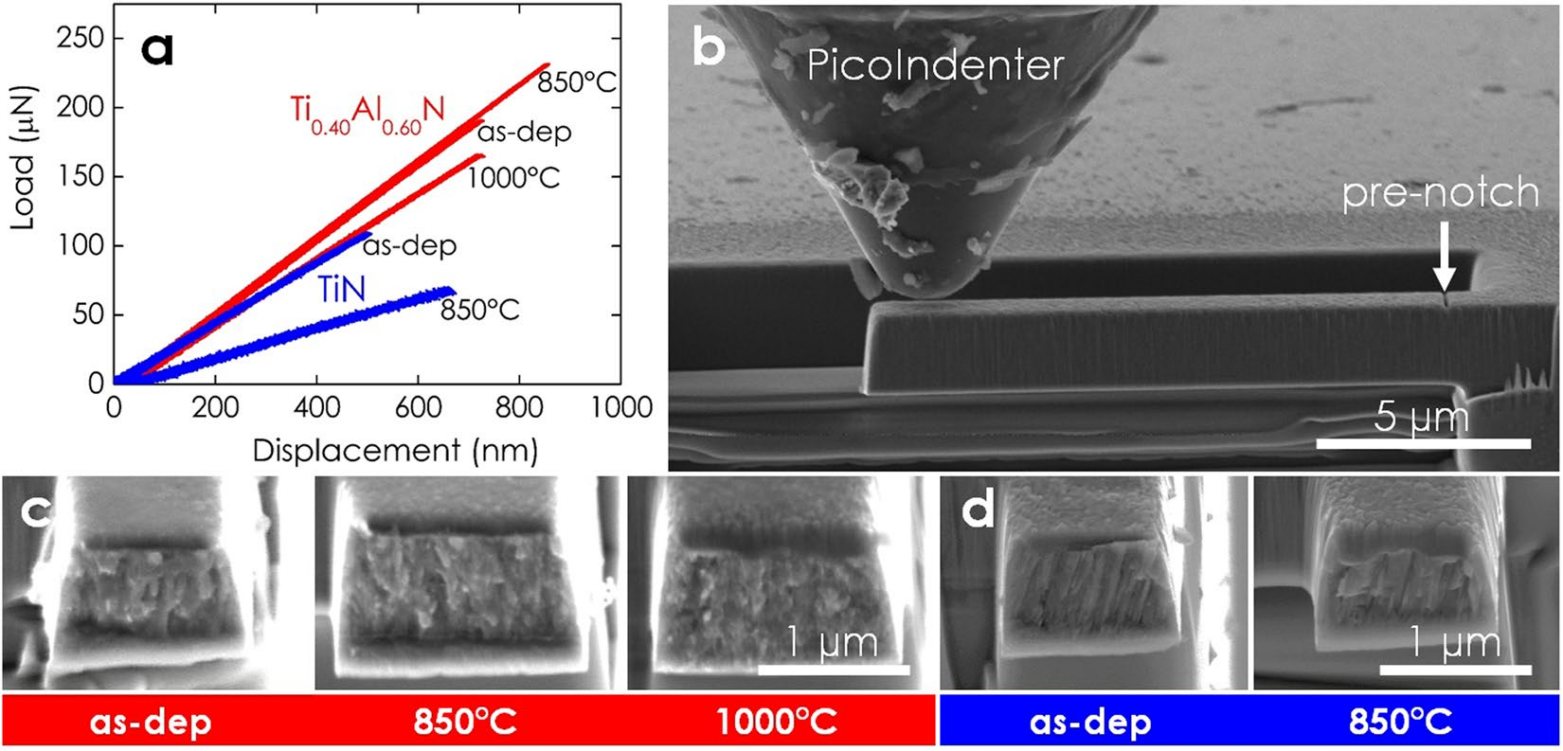
(**a**) Representative force–deflection curve of free-standing (*ex-situ*) annealed TiAlN cantilevers recorded during testing inside the scanning electron microscope. (**b**) The small dimension of the coating requires dedicated miniaturized micromechanical testing techniques. The image shows a scanning electron microscope image of the PicoIndenter tip approaching the pre-notched free-standing film cantilever. The cantilever is loaded until fracture. From simultaneous recorded load-deflection curves, the actual cantilever and pre-notch dimensions, and by applying fracture mechanics theory, the fracture toughness can be determined. (**c**) and (**d**) show the post-mortem fracture cross-sections (45° inclined view) of as-deposited and annealed TiAlN and TiN samples, respectively.

**Figure 5:**
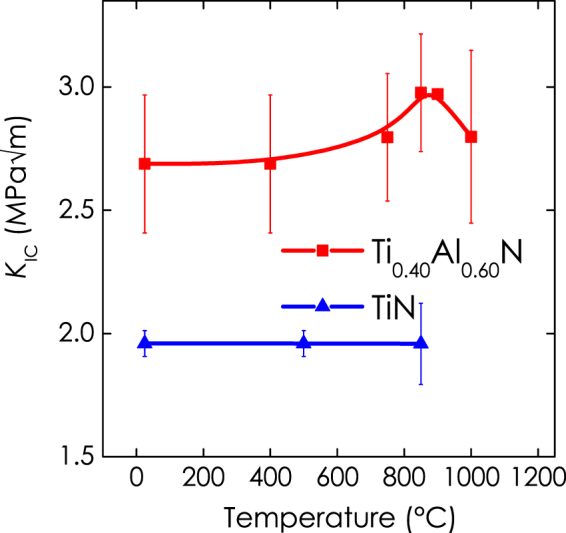
An increase in fracture toughness, *K*_IC_, is observed after 10 min vacuum annealing of the coatings at 850 and 900 °C (red curve). Such temperatures are typically reached in the application due to the friction between the coated cutting tool and the workpiece. In the case of TiN, where (spinodal) decomposition is absent, *K*_IC_ remains roughly constant when heated above the film deposition temperature.

Representative recorded force–deflection curves, given in Fig. 4a, show that Ti_0.40_Al_0.60_N and TiN deform in a linear manner, elastically during loading by a PicoIndenter until failure. No indications of plastic deformation are seen. (Please note that the actual cantilever dimensions, lever arms, and pre-notch depths differ from sample to sample. Hence, Fig. 4a, does not allow direct ranking of the samples with respect to their stiffness and fracture toughness). Figure 4b shows a typical free-standing cantilever. The substrate material had been removed by focused ion beam milling to avoid the influence of residual stresses and substrate interference. Scanning electron micrographs of the post-mortem fracture cross-sections, Fig. 4c,d, do not show discernible changes of the film morphology upon annealing. However, the structure of TiN (Fig. 4d) appears more columnar-grained in comparison with Ti_0.40_Al_0.60_N (Fig. 4c). The *K*_IC_ values, as calculated from the maximum load at failure, the actual pre-notch depth, and cantilever dimensions using a linear elastic fracture mechanics approach^27^, are presented in Fig. 5. The data suggest an increase in *K*_IC_ from 2.7 ± 0.3 MPa∙√m in the as-deposited state to 3.0 ± 0.01 MPa∙√m at 900 °C followed by a decreases to 2.8 ± 0.4 MPa∙√m at 1000 °C (red curve). The relative increase of ~11% in fracture toughness of Ti_0.40_Al_0.60_N is similar to the relative increase in hardness of ~9%. Please note, however, that strictly speaking the increase in fracture toughness is within statistical error. Interestingly, the pronounced decrease in hardness at 1000 °C due to wurtzite AlN formation is not observed for *K*_IC_, which —in agreement with the *H*/*E* criterion— only slightly decreases. Lower *K*_IC_ values of ~1.9 MPa∙√m are found for as-deposited and annealed TiN (blue curve).

**Figure 6:**
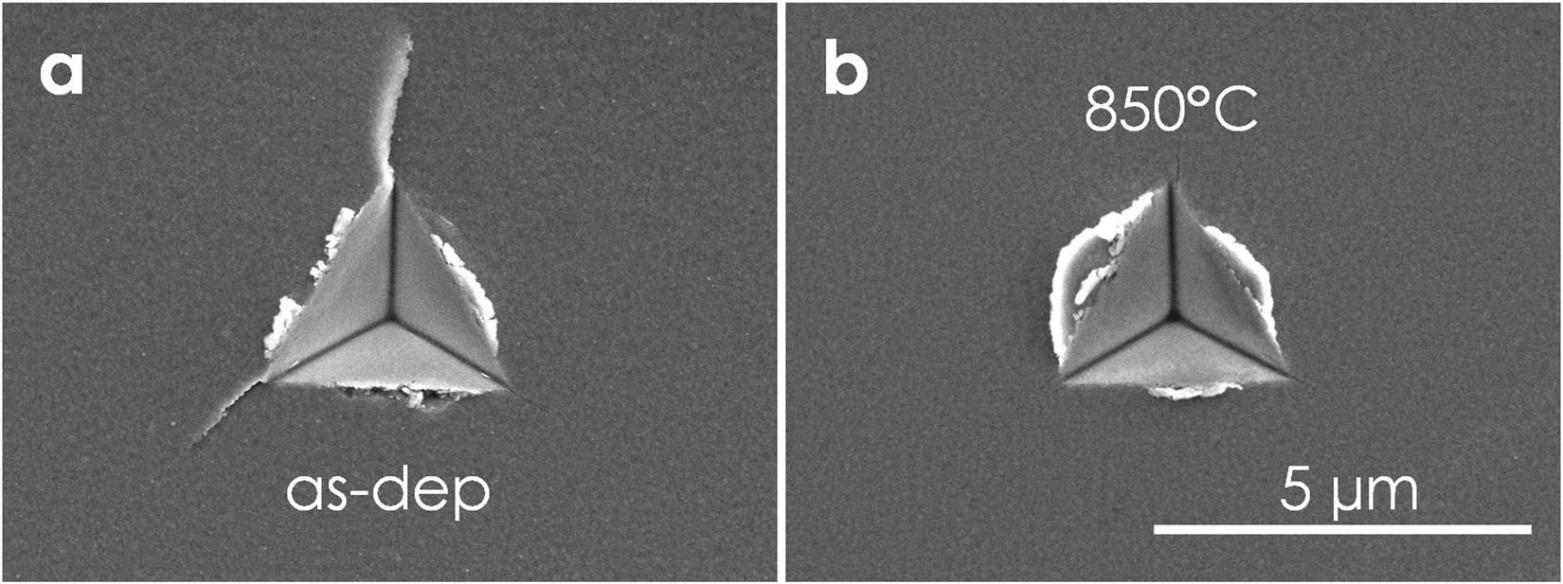
Cube corner nanoindentation experiments reveal aggravated (or even impeded) crack formation for annealed (**b**) as compared with as-deposited (**a**) Ti_1−x_Al_x_N coatings on sapphire substrates.

To qualitatively proof that *K*_IC_ increases upon annealing, we carried out independent cube corner nanoindenation experiments on coated Al_2_O_3_ $(1\bar{1}02)$ substrates. Scanning electron microscopy images of the indents show aggravated (or even impeded) crack formation for annealed Ti_1−x_Al_x_N samples as compared to the as-deposited counterpart, see Fig. 6. Please note that in the cube corner experiment, residual stresses (*e.g*., massive compressive residual stresses forming due to the cubic to wurtzite AlN phase transformation under volumes expansion) and the underlying substrate can influence the formation of cracks.

## Discussion

The structural evolution of supersaturated cubic Ti_1−x_Al_x_N upon annealing has been experimentally proven in the literature by atom probe tomography^11,28^, small angle X-ray scattering^29^, transmission electron microscopy^30^, and described by phase field simulations^30^: During the early stage, very few nanometer-sized B1 AlN- and TiN-rich domains form in a coherent manner (that is, the crystallographic orientation of the domains correspond to that of the Ti_1−x_Al_x_N parent grain). With progressive annealing time, the domains gain in size and the compositional variations become more pronounced, so that the modulation amplitudes (Ti- and Al-rich) become larger. If the annealing is continued for too long or performed at higher temperatures, coherency strains are relieved by misfit dislocations. Eventually, cubic structured AlN-rich domains transform into the softer but thermodynamically stable (first (semi) coherent then incoherent) hexagonal AlN. The cubic to hexagonal AlN phase transformation is associated with a large volume expansion of ~26%^16^.

Thermally-induced hardening effects in the TiAlN system have been attributed to coherency strains^9^. *Coherency strains* hinder the movement of dislocations^31^, as it is more difficult for dislocations to passage through a strained than a homogenous lattice. In addition, the coherent domains differ in their elastic properties due to the strong compositional dependent elastic anisotropy of Ti_1−x_Al_x_N^32^, which also hinders the dislocation motion and contributes to the hardness enhancement^32^.

The structural evolution observed in the present study is in line with the literature reports mentioned above. Additionally, we have evidenced severely distorted or inclined lattice planes and numerous defects (including stacking faults) in the hexagonal AlN phase by HRTEM investigations (Fig. 2). This could explain why the measured hardness at 900 °C is relatively high despite the presence of the “soft” hexagonal AlN phase, which is usually reported to deteriorate the hardness.

We have been able to show that besides age hardening effects, the fracture toughness increases upon annealing. Both properties show a similar relative increase of around 10% as compared to the as-deposited state and peak at the same temperature of 900 °C. This suggests that similar microstructural characteristics are responsible for the enhancement of the mechanical properties. We could demonstrate in an earlier study^3^ that a coherent nanostructure composed of alternating materials has the potential to enhance the fracture toughness for a certain bilayer period of a few nanometers. In the superlattice films, also *coherency strains* ^33,34^ and *variations in the elastic properties* are present. It should be mentioned, however, that in contrast to the hardness, the fracture toughness is not primarily governed by the hindrance of dislocation motion: the load-displacement data collected during the cantilever deflection experiments (Fig. 4a) suggest a linear elastic behavior until failure with no indications of plastic deformation.

In agreement with literature reports^21^, we found that cubic AlN forms preferentially at high diffusivity paths such as grain boundaries. If grain boundaries represent the weakest link where cracks preferentially propagate^35^, *grain boundary reinforcement* ^36^ has the potential to effectively hinder the crack propagation.

Another important mechanism for increased fracture toughness *is phase transformation toughening*, which is omnipresent in partially stabilized zirconia bulk ceramics^12^, for example. For Ti_1−x_Al_x_N coatings, the spinodally formed cubic structured AlN-rich domains represent the phase with the ability of a martensitic-like phase transformation from the metastable cubic structure to the stable wurtzite-type (w) variant. The associated volume expansion of ~26%^16^ slows down or closes advancing cracks, leading to a significant *K*_IC_ increase. Therefore, the evolution of *K*_IC_ with *T*_a_ of our Ti_0.40_Al_0.60_N coatings is not proportional to that of *H* with *T*_a_, especially at temperatures above 850 °C. The hardness significantly decreases for an increase of *T*_a_ from 950 to 1000 °C, as also the w-AlN formation significantly increases (please compare Figs. 1 and 3a), but at the same time, the fracture toughness *K*_IC_ only slightly decreases. The *K*_IC_ value of 2.8 ± 0.4 MPa∙√m after annealing at 1000 °C, is still above that of the as deposited state (with *K*_IC_ = 2.7 ± 0.3 MPa∙√m), whereas the hardness with *H* = 28 ± 2 GPa after annealing at 1000 °C is significantly below the as deposited value of 34 ± 1 GPa. Hence, effective other mechanisms are present in this type of material, especially when decomposition of the supersaturated matrix phase occurs and w-AlN based phases are able to form.

Note that in the chosen free-standing cantilever setup macro-stresses are relieved and thus do not contribute to the observed toughness enhancement. However, due to the extensive difference in the molar volume between cubic and wurtzite AlN, the thermally-induced formation of hexagonal AlN results in pronounced compressive stresses^17,37^ in the application where the coatings are firmly attached to a substrate/engineering component. Compressive stresses result in *apparent toughening* of Ti_1−x_Al_x_N, as the coating can withstand higher tensile stresses before cracks are initiated (the compressive stresses have to be overcome first before crack formation). The effect of compressive stresses on the fracture toughness is supposed to be much more pronounced than its influence on the hardness. This is why, in real application, the *K*_IC_ increase upon annealing is expected to be significantly larger than the *K*_IC_ enhancement found from free-standing micro-cantilever bending tests. This is reflected in the aggravated crack formation observed in the cube corner experiments, see Fig. 6.

As the ‘inherent’ fracture toughness enhancing effects are strongly connected with the spinodal decomposition, we anticipate that alloying^38–41^ and other concepts to modify the spinodal decomposition characteristics (formation of coherent cubic AlN domains at lower temperatures but delayed formation of the thermodynamically stable phase wurtzite AlN, different shape and size of cubic AlN domains) are applicable to optimize the self-toughening behavior. In general, alloying has the potential to enhance the inherent toughness by modifying the electronic structure and bonding characteristics^42,43^.

The peak in hardness and fracture toughness at 900 °C corresponds to spinodally decomposed TiAlN with fractions of hexagonal AlN as indicated by XRD (Fig. 1) and TEM (Fig. 2). The severely distorted hexagonal AlN with multiple stacking faults suggests that also nano-twinning might become a relevant mechanism. The presence of twins impedes dislocation motion and induces strengthening, but multiple twinning systems can also enhance ductility by acting as a carrier of plasticity^14^.

Based on our results we propose that the additional functionality of Ti_1−x_Al_x_N, *i.e*. the self-toughening ability at temperatures typical for many various applications, contributes to the outstanding performance of Ti_1−x_Al_x_N coatings in *e.g*., dry or high speed cutting.

## Methods

### Sample preparation

Ti_0.40_Al_0.60_N films were deposited in a lab-scale magnetron sputter system (a modified Leybold Heraeus Z400) equipped with a 3 inch powder-metallurgical processed Ti_0.50_Al_0.50_ compound target. Polished single crystalline Al_2_O_3_ $(1\bar{1}02)$ platelets (10 × 10 × 0.53 mm^3^) were chosen as substrate materials due to their high thermal stability, inertness and to avoid interdiffusion between film and substrate materials upon annealing up to 1000 °C. Before the deposition, the substrates (ultrasonically pre-cleaned in aceton and ethanol) were heated within the deposition chamber to 500 °C, thermally cleaned for 20 min and sputter cleaned with Ar ions for 10 min. The deposition was performed at the same temperature in a mixed N_2_/Ar atmosphere with a gas flow ratio of 4 sccm/6 sccm and a constant total pressure of 0.35 Pa by setting the target current to 1 A (DC) while applying a DC bias voltage of −50 V to the substrates. The films were grown to a thickness of about 1.8 µm with an average deposition rate of about 75 nm/min. The base pressure was below 5·10^–6^ mbar. TiN coatings of about 1.2 µm were synthesized by powering a 3 inch Ti cathode with 500 W within an N_2_/Ar gas mixture (flow ratio of 3 sccm/7 sccm, constant total pressure of 0.4 Pa) and applying a bias voltage of −60 V to the substrates. The deposition rate was about 13 nm/min.

Energy dispersive X-ray spectroscopy (EDXS) measurements of the films were performed with an EDAX Sapphire EDS detector inside a Philips XL-30 scanning electron microscope. Thin film standards characterized by elastic recoil detection analyses were used to calibrate the EDX measurements.

The films on Al_2_O_3_ were annealed in a vacuum furnace (Centorr LF22-2000, base pressure <3·10^−3^ Pa) at different maximum temperatures (*T*_a_) between 750 and 1000 °C using a heating rate of 20 °C min^−1^ and passive cooling. At *T*_a_, the temperature was kept constant for 10 min.

Structural investigations of coated Al_2_O_3_ substrates were performed by X-ray diffraction in symmetric Bragg-Brentano geometry using a PANalytical X’Pert Pro MPD diffractometer (Cu-K_α_ radiation).

Cross-sectional TEM specimens were prepared using a standard TEM sample preparation approach including cutting, gluing, grinding and dimpling. Finally, Ar ion milling was carried out. A JEOL 2100 F field emission microscope (200 kV) equipped with an image-side C_S_-corrector with a resolution of 1.2 Å at 200 kV was used. The aberration coefficients were set to be sufficiently small, *i.e*. C_S_ ~ 10.0 μm. The HRTEM images were taken under a slight over-focus. The HRTEM images were carefully analysed using Digital Micrograph software.

### Micromechanical testing

The mechanical properties, hardness and indentation modulus, were measured using a UMIS nanoindenter equipped with a Berkovich tip. At least 30 indents per sample, with increasing loads from 3 to 45 mN were performed. The recorded data were evaluated using the Oliver and Pharr method^44^. To minimize substrate interference, only indents with indentation depths below 10% of the coating thickness were taken into account. The cube corner experiments were carried with the UMIS nanoindenter using a peak indentation load of 150 mN. The high load needed to create cracks resulted in indentation depths of about 1.3 µm in the cube corner experiment.

The fracture toughness was determined from micromechanical cantilever bending tests of free-standing film material. As-deposited and annealed coated Al_2_O_3_ samples were broken and their cross-sections carefully polished. The substrate material was removed by Focused Ion Beam (FIB) milling perpendicular to the film growth direction using a FEI Quanta 200 3D DBFIB work station. Then the sample holder was tilted 90° and cantilevers were milled perpendicular to the film surface. The cantilever dimensions of ∼*t* × *t* × 6*t* μm^3^, with *t* denoting the film thickness, were chosen based on guidelines reported in Brinckmann *et al*.^45^ For the final milling step, the ion beam current was reduced to 500 pA, the initial notch was milled with 50 pA. To circumvent the problem of a finite root radii on the fracture toughness measurements, bridged notches according to Matoy *et al*.^27^ were used (the notch length was chosen to be ∼0.75*t*).

The micromechanical experiments were performed inside a scanning electron microscope (FEI Quanta 200 FEGSEM) using a PicoIndenter (Hysitron PI85) equipped with a spherical diamond tip with a nominal tip radius of 1 μm. The micro-cantilever beams were loaded displacement-controlled with 5 nm/s with the loading axis perpendicular to the film surface. Per annealing temperature at least 3 tests were conducted. The fracture toughness, *K*_IC_, was determined using linear elastic fracture mechanics according to the formula given in ref.^27^:

1 $${K}_{IC}=\frac{{F}_{max}L}{B{W}^{3/2}}f\,(\frac{a}{w})$$

with $f\,(\frac{a}{W})=1.46+24.36\ast (\frac{a}{W})-47.21\ast {(\frac{a}{W})}^{2}+75.18\ast {(\frac{a}{W})}^{3}$. In the equation, ${F}_{max}$ denotes the maximum load applied, *L* the lever arm (distance between the notch and the position of loading), *B* the width of the cantilever, *W* the thickness of the cantilever, and *a* the initial crack length (measured from the post mortem fracture cross-sections).
